# A newly isolated *Trichoderma Parareesei* N4-3 exhibiting a biocontrol potential for banana fusarium wilt by Hyperparasitism

**DOI:** 10.3389/fpls.2023.1289959

**Published:** 2023-10-24

**Authors:** Weiqiang Long, Yufeng Chen, Yongzan Wei, Junting Feng, Dengbo Zhou, Bingyu Cai, Dengfeng Qi, Miaoyi Zhang, Yankun Zhao, Kai Li, Yong-Zhong Liu, Wei Wang, Jianghui Xie

**Affiliations:** ^1^ National Key Laboratory of Tropical Crop Breeding, Institute of Tropical Bioscience and Biotechnology, Chinese Academy of Tropical Agricultural Sciences, Haikou, Hainan, China; ^2^ National Key Laboratory for Germplasm Innovation and Utilization of Horticultural Crops, College of Horticulture and Forestry Sciences, Huazhong Agricultural University, Wuhan, Hubei, China

**Keywords:** banana fusarium wilt, biological control, *Trichoderma*, enzyme, hyperparasitism

## Abstract

Banana Fusarium wilt caused by *Fusarium oxysporum* f. sp. *cubense* tropical race4 (*Foc* TR4) is one of the most destructive soil-borne fungal diseases and currently threatens banana production around the world. Until now, there is lack of an effective method to control banana Fusarium wilt. Therefore, it is urgent to find an effective and eco-friendly strategy against the fungal disease. In this study, a strain of *Trichoderma* sp. N4-3 was isolated newly from the rhizosphere soil of banana plants. The isolate was identified as *Trichoderma parareesei* through analysis of *TEF1* and *RPB2* genes as well as morphological characterization. *In vitro* antagonistic assay demonstrated that strain N4-3 had a broad-spectrum antifungal activity against ten selected phytopathogenic fungi. Especially, it demonstrated a strong antifungal activity against *Foc* TR4. The results of the dual culture assay indicated that strain N4-3 could grow rapidly during the pre-growth period, occupy the growth space, and secrete a series of cell wall-degrading enzymes upon interaction with *Foc* TR4. These enzymes contributed to the mycelial and spore destruction of the pathogenic fungus by hyperparasitism. Additionally, the sequenced genome proved that strain N4-3 contained 21 genes encoding chitinase and 26 genes encoding *β*-1,3-glucanase. The electron microscopy results showed that theses cell wall-degrading enzymes disrupted the mycelial, spore, and cell ultrastructure of *Foc* TR4. A pot experiment revealed that addition of strain N4-3 significantly reduced the amount of *Foc* TR4 in the rhizosphere soil of bananas at 60 days post inoculation. The disease index was decreased by 45.00% and the fresh weight was increased by 63.74% in comparison to the control. Hence, *Trichoderma parareesei* N4-3 will be a promising biological control agents for the management of plant fungal diseases.

## Introduction

Banana is the important staple and cash crops in tropical and subtropical regions. About 500 million people depend on bananas as a staple food in the world (Lozano-Durán et al., 2022). Meanwhile global banana exports have been largely on an upward trend. The world banana trade reached $28.963 billion US dollars in 2022 (Zou and Fan, 2022). The domestication and asexual reproduction techniques of most seedless edible banana varieties limit the genetic variation of bananas, ultimately leading to their susceptibility to pests and diseases (Perrier et al., 2011).

Banana Fusarium wilt, caused by *Fusarium oxysporum* f. sp. *cubense* (*Foc*), is the most destructive and threatening soil-borne fungal disease in banana production (Chen et al., 2022). The causal agent is subdivided into four subspecies depending on the infected banana species (Dita et al., 2018). One of these pathogenic fungi named *Fusarium oxysporum* f. sp. *cubense* tropical race 4 (*Foc* TR4) can infect almost all banana varieties (Dita et al., 2018; García-Bastidas, 2019). Notably, *Foc* TR4 spores are able to tolerate harsh environmental conditions and survive in soil for more than 20 years (Qi et al., 2021). They are capable of spreading on non-host species by soil, irrigation or agricultural equipment (Ploetz, 2015a). To date, there are no commercially available banana varieties that are resistant to *Foc* TR4. In addition, *Foc* TR4 is insensitive to some fungicides and long-term application of chemical pesticides caused a bad pollution on soil ecology (Cannon et al., 2022). In contrast, microbial fungicides have the ability to control pathogenic fungi and do not damage soil ecology. Therefore, the application of microorganisms to control pathogenic fungi has good prospects.

*Trichoderma* spp. present in soil and plants are diverse and easy to pure cultivation (Guo et al., 2019; Savani et al., 2021). *Trichoderma* species are fast-growing fungus that compete with pathogenic fungi for nutrition and living space, thereby limiting the growth of pathogenic fungi. They can parasitize the hyphae of pathogenic fungi and produce cell wall-degrading enzymes, ultimately leading to the death of the pathogenic fungi. Additionally, *Trichoderma* can also inhibit the growth of plant pathogens by producing secondary metabolites and volatile organic compounds that have antagonistic activity (Harni et al., 2020; Lv et al., 2023). For instance, the active metabolite gliotoxin, isolated from the culture broth of *Trichoderma virens* HZA14, was found to completely inhibit the growth of *Phytophthora capici* mycelium when added to PDA at a concentration of 5.0 μg/mL (Tomah et al., 2020). Rajani et al. found that *Trichoderma longibrachiatum* MK751759 can produce volatile organic compounds to inhibit the growth of *Sclerotium rolfsii CSR* (Rajani et al., 2021). In addition, *Trichoderma* has been shown to stimulate systemic induced resistance in host plants (Mukherjee et al., 2013; Sood et al., 2020). Although a large number of studies on *Trichoderma* have been reported (Tomah et al., 2020; Díaz-Gutiérrez et al., 2021; Tamizi et al., 2022), the inhibitory mechanism of different *Trichoderma* on pathogens still needs to be elucidated. Therefore, it is necessary to isolate *Trichoderma* strains with high antagonistic activity and characterize their antagonistic mechanism.

In this study, an antagonistic strain N4-3 against *Foc* TR4 was isolated from banana plantation soil. Morphological identification and multigene association analysis were used to determine its taxonomic characteristics. We further evaluated its broad-spectrum antifungal activity against various plant pathogenic fungi. The hyperparasitism of strain N4-3 was confirmed by observing the dual culture region using scanning electron microscopy. In order to elucidate the antifungal mechanism of strain N3-4, the cell wall-degrading enzymes secreted by the isolate were extracted and identified. And further testing was conducted to investigate the effects of enzymes on the mycelium, spores, and cellular ultrastructure of the pathogenic fungus. Genomic analysis revealed the presence of a large number of genes encoding chitinases and *β*-1,3-glucanase in the genome of strain N4-3. A pot experiment was carried out to further investigate the biocontrol efficiency of strain N4-3 against *Foc* TR4. The results will provide additional selection for agricultural biocontrol agents and biofertilizers.

## Materials and methods

### Isolation of *Trichoderma* spp.

Soil samples was collected from rhizosphere of a banana plantation without symptom of banana fusarium wilt for more than ten years in Lingao County, Hainan Province, China (19°45’3 “N, 109°55’17 “E). The collected samples were transported to the laboratory in a sterile plastic bag and stored at 4°C. *Trichoderma* were isolated from soil samples by a gradient dilution method (Rahman et al., 2023). Briefly, 5 g of fresh soil was added to 45 mL of sterile distilled water, incubated at 28°C with shaking at 180 r/min for 30 min, and then diluted with sterile water to 10^-1^~10^-3^. 100 µL of diluted soil suspension was poured onto the Rose Bengal Agar (RBA) (5.0 g peptone, 10.0 g glucose, 1.0 g dipotassium hydro gen phosphate, 0.5 g magnesium sulfate, 0.033 g Bengal Red, 0.1 g chloramphenicol, 20.0 g agar in one liter water) was spread on the plate using an applicator. Subsequently, the plates were placed at 28°C and incubated for 3-5 days. Single colony on the selection medium was transferred to the potato dextrose agar medium (PDA) for purification. The purified strains were stored at 4°C for the following study.

### Antifungal activity screening of *Trichoderma* against *Foc* TR4

The isolate with the highest antifungal activity against *Foc* TR4 was screened by a dual culture method (Tian et al., 2016). Mycelial discs (5 mm in diameter) of *Trichoderma* were placed on the edge of PDA plates and the same size mycelial discs of *Foc* TR4 were placed on the opposite side. A mycelial disc of *Foc* TR4 alone was used as a control. Plates were incubated at 28°C for 7 days. The radius of *Foc* TR 4 colonies on the dual culture plates was determined. The inhibition rate (MI) was calculated using the following equation: MI = [(R_1_-R_2_)/R_1_] ×100, where R_1_ and R_2_ represented the radius of *Foc* TR4 colonies in the control and the treatment groups, respectively. Based on inhibition rate against *Foc* TR4, an isolate, labeled as strain N4-3, with strong antifungal activity was selected for the following study.

### Morphological, physiological and biochemical characteristics of strain N4-3

Mycelial discs of strain N4-3 from actively growing colonies were placed on PDA plates, incubated at 28°C for 48-72 h. Morphological characteristics of mycelium and spores were observed using biomicroscope (CellcutPlus, MMI, Germany) and scanning electron microscope (SEM, SIGMA Field Emission Scanning Electron Microscope). The physiological and biochemical parameters of strain N4-3 were determined, including resistance to pH and utilization of carbon and nitrogen sources (Wei et al., 2020).

### Phylogenetic analysis

DNA from fresh mycelium was extracted using the Fungal Genome Rapid Extraction Kit (Aidlab, China). The primers for RNA polymerase II subunit (rpb2) were fRPB2-5f (GAYGAYMGWGATCAYTTYGG) and fRPB2-7cr (CCCATRGCTTGTYYRCCCAT). The primers for translation elongation factor 1 *α*(tef1) were EF1 (ATGGGTAAGGARGACAAGAC) and EF2 (GGARGTACCAGTSATCATGTT) (Liu et al., 1999; Kothe et al., 2016; Cai and Druzhinina, 2021). The amplified PCR products were sequenced using a Sanger-based automated sequencer (Applied Biosystems). Sequences were edited by DNAMAN. Reference sequences were analyzed by nucleotide BLAST searches in the GenBank database (**Supplementary Table 1**). Phylogenetic trees were constructed by using two locus combinations of the *TEF1* and *RPB2* datasets. The gene sequences were aligned with MAFFT and the generated sequences were edited by Gblocks to remove ambiguously aligned positions and divergent regions prior to phylogenetic analysis. Two locus data were pooled and analyzed using the maximum likelihood method in MEGA Version 7.0.

### Sequencing and annotation of the strain N4-3 genome

Genomic DNA was extracted from strain N4-3. Libraries were constructed using the Hieff NGS^®^ MaxUp II DNA Library Preparation Kit for Illumina^®^ and quantified using the Thermo Qubit 4.0 Fluorescence Quantification Instrument Q33226 (ThermoFisher). Sequencing was performed on the Illumina High-Throughput Sequencing Platform (HiSeq). Quality of raw sequencing data was assayed by FastQC. Sequence correction was performed using PrInSeS-G to correct for editing errors and insertion deletion of small fragments during splicing. Genetic components such as genes, tRNAs, rRNAs were predicted using GeneMark, etc. The repetitive sequences were identified using Repeat Masker (Koren et al., 2017). CRISPR prediction analysis was performed using CRT. Gene protein sequences were aligned with multiple databases such as CDD, KOG, COG, NR, NT, PFAM, Swissprot and TrEMBL. GO and KEGG annotation was analyzed according to the previous methods (Wei et al., 2020).

### Determination of the broad-spectrum antifungal activity of strain N4-3

To evaluate the broad-spectrum antifungal activity of strain N4-3, ten phytopathogenic fungi were selected including *C. gloeosporioides* (ATCC 58222), *C. gloeosporioides* (ATCC16330), *C. fragariae* (ATCC 58718), *C. lunata* (ATCC 42011), *F. graminearum Sehw* (ATCC MYA-4620), *C. musae* (ATCC 96167), *F. oxysporum f.* sp. *cucumerinum* (ATCC 204378), *C. acutatum* (ATCC56815), *C. gloeosporioides* (ATCC MY A-456), and *C. fallax* (ATCC 38579). A 5 mm mycelial discs of *Trichoderma* were placed on the edge of PDA plates. The same size discs of various pathogenic fungi were placed on the opposite side. The pathogenic mycelial discs alone were used as a control. All experiments were repeated in triplicate.

### Interaction observation between strain N4-3 and *Foc* TR4

The dynamics of strain N4-3 and *Foc* TR4 interactions was observed using a dual culture method (Damodaran et al., 2020). The discs were punched at the colonial edge of *Trichoderma* and *Foc* TR4, respectively, and placed on the PDA plates at 28°C for incubation. The growth was recorded at 12 h intervals. After successful fungal superparasitism on the pathogen, the dual culture areas were observed using the UItra-depth-of-field Three-dimensional Microscope system (VHX-600, Kerns). Mycelial region of *Foc* TR4 on the dual culture plate were selected and fixed in 2.5% of glutaraldehyde for 12 h. The samples were dehydrated with a gradient ethanol (30, 50, 70, 80, 90, 95, 100%) for 2-3 min each time. The samples were freeze-dried, and then observed by scanning electron microscopy (SEM).

### Determination of antifungal activity of extracts and volatile organic compounds of strain N4-3

The metabolites of strain N4-3 were extracted according to Zhang et al. (Zhang et al., 2021). Briefly, strain N4-3 was incubated in PDB at 28°C and 180 r/min for 7 days. The culture broth was mixed with an equal volume of anhydrous ethanol and shaken at 150 rpm for 3 days. After filtration through the Whatman No.1 filter, the extract was evaporated using a rotary evaporator. The dried extracts were dissolved into saturated solution using sterile water. The extracts solution was used to test antimicrobial activity by an agar diffusion method.

Antifungal activity of volatile organic compounds produced by strain N4-3 was tested according to Rajani et al. (Rajani et al., 2021). Mycelial discs (5 mm in diameter) of strain N4-3 and *Foc* TR4 were picked from the edge of colonies and placed on two separate plates containing PDA. The two plates were placed against each other. To prevent cross-contamination, a sterile cellophane layer was added between the two petri dishes. Only mycelial discs of *Foc* TR4 were used as a control. The inhibition rate was measured and calculated after 7 days.

### Crude enzyme solution extraction of strain N4-3

Strain N4-3 was incubated in the PDB medium at 28°C and 180 r/min with shaking for 7 days. The culture solution was filtered through filter paper to collect the filtrate. 100 mL of the filtrate was then added to a 250 mL conical flask. Ammonium sulfate was added to prepare a mixed solution with varying saturations (10%, 20%, 30%, 40%, 50%, 60%, 70%, 80%, 90% and 100%) and left overnight at 4°C (Xu et al., 2022). At least three replicates of each experiment were performed. The mixture was then centrifuged at 10,000 r/min for 10 min at 4°C, and the supernatant was discarded. The precipitates were suspended in 2 mL of phosphate buffer (pH 7.2-7.4) to obtain crude enzyme solution for chitinase and *β*-1,3-glucanase.

### Inhibition efficiency of crude enzyme solution on mycelial growth of *Foc* TR4

The crude enzyme solution was freeze-dried to powder. Different concentrations (400, 200, 100, 50 and 25 mg/mL) of crude enzyme were prepared using PBS. Inhibition efficiency was tested as described by Mohiddin et al. (2021). After filtering through a membrane to remove bacteria, 200 µL of the crude enzyme solution were evenly poured to the PDA medium and inoculated with *Foc* TR4 mycelial blocks. The crude enzyme solution after high-temperature inactivation was used as a control. At least three replicates were set for each concentration. The inhibition efficiency was calculated by measuring the colony diameter after 5 days. A toxicity regression equation was established as a linear regression using a least squares method. The EC_50_ value was calculated from the toxicity regression equation (Wei et al., 2020).

### Inhibition of crude enzyme solution on spore germination of *Foc* TR 4

The germination rate of *Foc* TR4 spores was measured as the description of Li et al. (2021) with a minor modification. Firstly, 20 µL of spore suspension (1.0 × 10^6^ CFU/mL) was mixed with an equal volume of crude enzyme solution at a final concentration of 1× EC_50_. The mixture was then added to a concave slide. The same concentration of heat-inactivated crude enzyme solution was used as a control. The spores were incubated at 28°C for 24 h, and 100 spores on each slide were observed using a light microscope. The spore germination rate was used to evaluate the inhibition efficiency. All experiments were performed in triplicates.

### Effect of crude enzyme solution on the growth of *Foc* TR4 mycelia

The mycelial discs of *Foc* TR4 were inoculated in the center of the PDA plate, and four sterilized coverslips were placed equidistantly around the plate. The plate was incubated at 28°C until the mycelia of *Foc* TR4 covering the whole coverslips. Subsequently, 100 µL of crude enzyme solution (1×EC_50_) filtered through a syringe filter and covered with mycelia on the coverslips. The plates were gently shaken to ensure that the enzyme solution covered the entire slide. Two coverslips per plate were treated with the crude enzyme solution. Other two coverslips were treated with the same concentration of heat-inactivated crude enzyme solution as a control. After one day of incubation, the morphological changes of the mycelia were observed under a light microscope.

### Effect of crude enzyme solution on spore morphology of *Foc* TR4

To prepare a spore suspension of *Foc* TR4, 100 µL of spore suspension (1.0 × 10^6^ CFU/mL) was pipetted onto a coverslip, followed by the addition of 100 µL of crude enzyme solution (2 × EC_50_) (Chen et al., 2018). The same concentration of heat-inactivated crude enzyme solution was used as a control. The spores were incubated at 28°C for 24 h. After fixation, changes of spore morphology were observed using SEM.

### Effect of crude enzyme solution on the ultrastructure of *Foc* TR4 cells

Mycelial discs were inoculated at the center of PDA plates. After two days at 28°C, the peripheral mycelia of colonies were treated with a crude enzyme solution (1 × EC_50_). The control group was treated with the same concentration of heat-inactivated crude enzyme solution. After one day of incubation, samples were prepared according to Yun et al. (2021). The ultrastructure of *Foc* TR4 cells was observed with using a transmission electron microscope (TEM, JEM-1400Flash, Hitachi, Ltd., Tokyo, Japan).

### Pot experiment

Banana seedlings (*Musa acuminata* L. AAA genotype cv. Cavendish) with the height of 8-10 cm were transplanted into plastic pots (8 cm × 8 cm) containing 1000 g of sterilized soil. The plants were then placed in a greenhouse at 28°C and a relative humidity of 70-80%. *Foc* TR4 expressing the GFP gene (GFP-*Foc* TR4) was provided by the Institute of Environment and Plant Protection, Chinese Academy of Tropical Agricultural Sciences, Haikou, China. Three treatments were set, including Blank (sterile water), Control (positive control, inoculated with GFP-*Foc* TR4) and Treatment (1.0 × 10^7^ CFU/mL of strain N4-3 fermentation broth and 1.0 × 10^7^ CFU/mL of GFP-*Foc* TR4). 100 mL of mixture was added to the roots of banana seedlings (Jing et al., 2020). Banana seedlings were treated at 7 days intervals. Roots were selected at 60 days post inoculation (dpi) and sections were made using a manual method (Jing et al., 2020). *Foc* TR4 infection was observed with a laser scanning confocal microscope (ZEISS, LSM800, Germany). The maximum photosynthetic efficiency, relative chlorophyll content, leaf area, leaf thickness, stem thickness, plant height, fresh weight and index of photosynthetic system II were also measured. Inter-root soil was collected for *Foc* TR4 quantification as our previous description (Chen et al., 2018). The number of *Foc* TR4 colony-forming units (CFU/g) was determined using the inter-root soil suspension gradient dilution method. Each experiment was replicated in triplicate. Ten plants were used in each replication.

### Statistical analysis

The data were analyzed using the SPSS software (Version 23.0). All experiments were performed in triplicate. Significant difference (p < 0.05) was determined using the Duncan’s multiple extreme difference test. The results obtained from three independent experiments were expressed as a mean ± standard deviation.

## Results

### Isolation and screening of *Trichoderma* spp.

**Figure 1:**
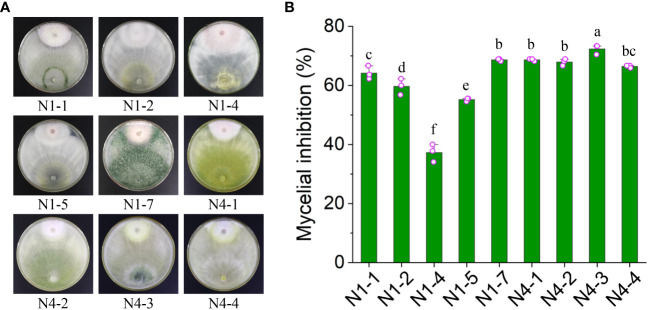
Inhibition efficiency of nine isolated fungi against *Foc* TR4. **(A)** Dual culture of nine fungal strains with *Foc* TR4. **(B)** Inhibition efficiency of nine fungal strains against *Foc* TR4. Different lowercase letters represented the significant difference (P<0.05).

To screen *Trichoderma* spp. exhibiting antifungal activity against *Foc* TR4, the rhizosphere soil was selected from banana plantations without disease symptom for ten years. A double incubation experiment was performed. Nine members of *Trichoderma* spp. were obtained and incubated with *Foc* TR4 at 28°C for 7 days. The colonial radius of *Foc* TR4 was measured in both the treatment and control groups (**Figure 1A**). The inhibition efficiency of each strain was calculated. The results showed that strain N4-3 exhibited the highest inhibition activity at 72.37 ± 1.67%, whereas N1-4 displayed the lowest inhibition efficiency at 37.29 ± 2.98%. The inhibition rate of strain N4-3 was significantly higher than that of other strains (**Figure 1B**). Therefore, strain N4-3 was selected for the following experiment.

### Identification of strain N4-3

**Figure 2:**
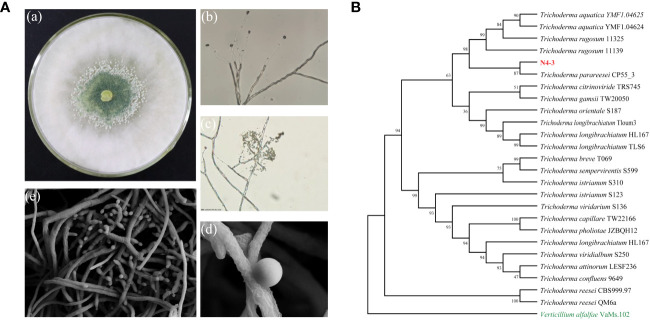
Morphological observations and phylogenetic analysis of strain N4-3. **(A)** Morphological observation of strain N4-3. a.Morphological characterization of N4-3 grown on top of potato dextrose agar medium, b-c. Morphological characteristics of spores and hyphae of N4-3 were observed using a biomicroscope, d-e Morphological characteristics of spores and hyphae of N4-3 were observed using a scanning electron microscope. **(B)** Phylogenetic tree generated by maximum likelihood analysis based on the tandem sequence alignment of two genes (*tef1* and *rpb2*).

After 48 h of growth on the PDA medium, strain N4-3 exhibited abundant aerial mycelia. The conidiophores were densely packed and appeared white or green in color (**Figure 2A**). The conidia were ellipsoidal in shape and grew along the main axis, with terminal thick-walled conidia that were globose to subglobose in shape (**Figure 2A**). Strain N4-3 produced a yellow pigment on the PDA medium (**Supplementary Figure 1C**). The results of physiological and biochemical tests indicated that strain N4-3 exhibited tolerance to pH ranging from 5 to 9. Additionally, strain N4-3 demonstrated the ability to utilize all 16 tested carbon sources. Among the tested nitrogen sources, strain N4-3 could utilize eight sources, except for L-arginine (**Supplementary Table 2**).

The sequencing of PCR products was assembled using DNAMAN software. After alignment using MAFFT and editing with Gblocks, the length of *tef1* and *rpb2* were 1127 bp and 1074 bp, respectively. To analyze the evolutionary relationship of strain N4-3, *tef1* and *rpb2* were used for constructing the phylogenetic tree by alignment of the selected 25 type strains. The results revealed that strain N4-3 clustered with *Trichoderma parareesei* CP55_3, forming a distinct evolutionary branch with 87% of ML-BS (**Figure 2B**). Combining with the morphological characteristics, strain N4-3 was identified as *Trichoderma parareesei*.

### Genome assembly and annotation

**Figure 3:**
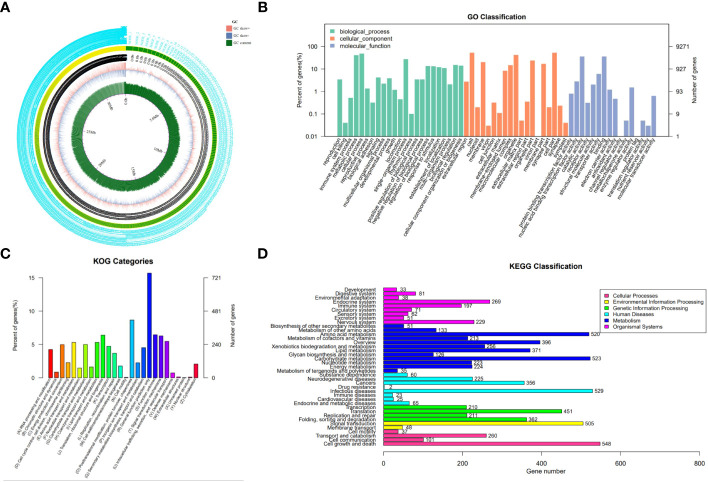
Genomic information of strain N4-3 and functional annotation of predicted genes. **(A)** Gene circle map of strain N4-3. **(B)** Histogram of GO annotation. The horizontal axis was the secondary classification of GO and the vertical axis was the number of genes. Different colors represented different orthologs. **(C)** Histogram of KOG classification. Each color on the horizontal axis represented a functional classification of KOG and the vertical axis was the number of genes. **(D)** Classification of KEGG Pathway. The vertical axis was the name of the metabolic pathway and the vertical axis was the number of genes.

By sequencing and assembling, the genome size of strain N4-3 was 32,339,192 bp. The GC content was 54% and repetitive sequences accounted for 2.08%. A total of 9,271 genes were predicted for the strain N4-3 genome, with an average sequence length of 1,522 bp (**Figure 3A**; **Supplementary Table 3**). Among these genes, 6,320 (68.17%), 4,806 (51.84%), and 3261 (35.17%) were annotated using GO, KOG and KEGG, respectively (**Supplementary Table 4**). The annotated genes were classified according to the GO terms in the three categories of Biological Process, Cellular Component and Molecular Function, (**Figure 3B**). In KOG, the pathways with high number of annotated genes were translation, ribosome structure and biogenesis (6.39%), followed by amino acid transport and metabolism (5.31%) and energy production and transformation (4.97%) (**Figure 3C**). The metabolic pathways of KEGG were classified based on the linkage between KO and pathway. The pathways were divided into five branches: cellular processes, environmental information processing, genetic information pathways, metabolism and organismal systems (**Figure 3D**).

### Strain N4-3 exhibiting the broad-spectrum antifungal activity

**Figure 4:**
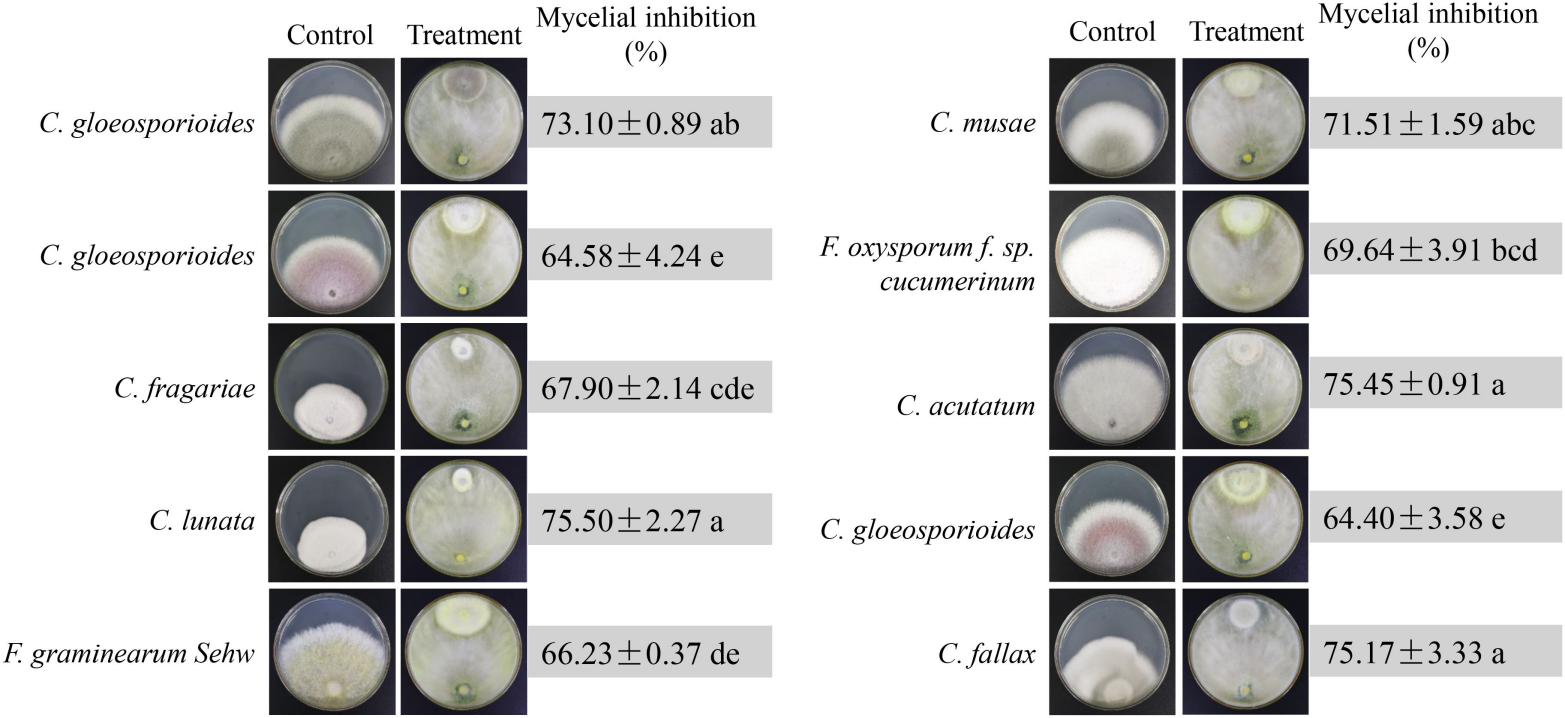
Broad-spectrum inhibition efficiency test of N4-3 against 10 phytopathogenic fungi. Data are expressed as mean ± standard deviation. Different lowercase letters indicate significant differences at P < 0.05 in Duncan’s multiple range test.

Ten phytopathogenic fungi were selected to test the broad-spectrum antagonistic potential of strain N4-3. The antifungal activity was measured after one week of dual incubation. Strain N4-3 exhibited different inhibition ability against different pathogenic fungi (**Figure 4**). By constrast, strain N4-3 had a strong inhibitory activity on the mycelial growth of *C. lunata* (ATCC 42011), *C. acutatum* (ATCC56815) and *C. fallax* (ATCC 38579), resulting in a reduction in colony growth diameters of 75.45 ± 0.91, 75.45 ± 0.91, and 75.17 ± 3.33, respectively. The least inhibitory activity was observed against *C. gloeosporioides* (ATCC MYA-456), with a reduction in colony growth diameter of 64.40 ± 3.58 (**Figure 4**). These results indicated that strain N4-3 possessed a broad-spectrum antagonistic ability against phytopathogenic fungi.

### Interaction of strain N4-3 with *Foc* TR4

**Figure 5:**
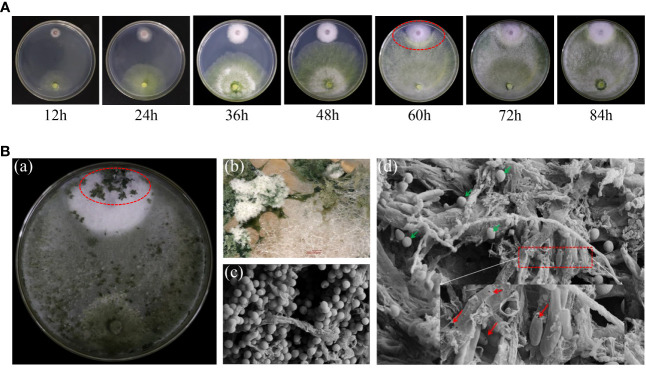
Interaction of strain N4-3 with *Foc* TR4. **(A)** Dynamic process of the interaction between strain N4-3 and *Foc* TR4. The red elliptical region indicated the appearance of the inhibition circle. **(B)** Dual culture of strain N4-3 and *Foc* TR4 was carried out for 10 days (a). Spore clusters of strain N4-3 appeared in the *Foc* TR4 colony area (b). The dual culture area was observed using SEM (c-d). The green arrow showed the spores of strain N4-3 and the red arrow showed the mycelia and spores of *Foc* TR4.

We further detected the dynamic interaction between strain N4-3 and *Foc* TR4. Observation was recorded at 12-hour intervals during the dual incubation. The results showed that two strains started to come into contact at 48 h and show inhibition circles at 60 h (**Figure 5A**). Numerous distinct clusters of green *Trichoderma* spores appeared in the colony area of *Foc* TR4 on 10th day. The parasitic area was observed using a stereomicroscope. Mycelia and spores of strain N4-3 attached to the mycelia of *Foc* TR4, resulting in the lysis of the pathogenic fungal mycelia (**Figure 5B**). Additionally, a large number of *Trichoderma* spores were observed in the pathogenic area, and gaps were also found in the pathogenic fungal mycelia and spores (**Figure 5B**). Therefore, strain N4-3 was able to destroy the complete structure of mycelia and spores of *Foc* TR4.

#### Determination of antifungal components of strain N4-3

**Figure 6:**
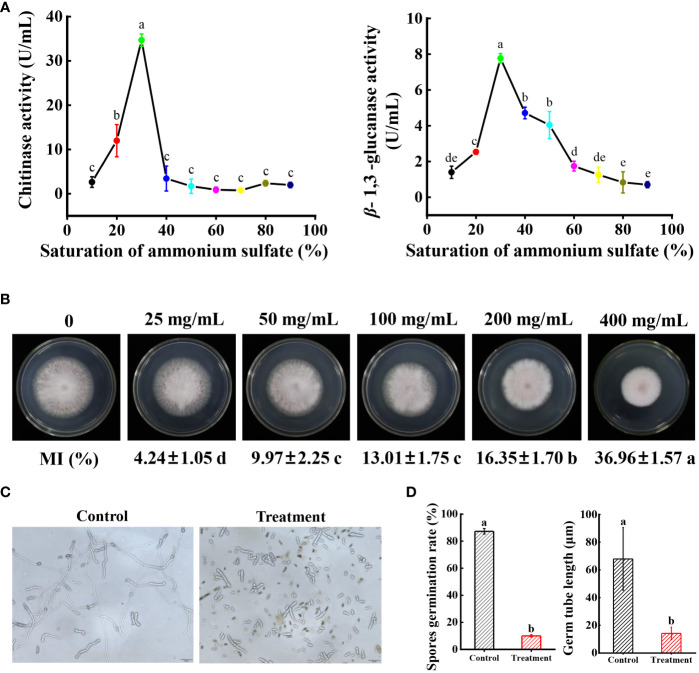
Determination of *Foc* TR4 antagonism efficiency of crude enzyme solution. **(A)** Determination of chitinase activity and *β*-1,3-glucanase activity in crude enzyme solutions extracted with different saturations of ammonium sulfate. **(B)** Inhibition efficiency of *Foc* TR4 by different concentrations of crude enzyme solution. Data are expressed as mean ± standard deviation. **(C)** Spores were treated with EC_50_ crude enzyme solution, incubated for 1 day, and then observed under a biological microscope. **(D)** Statistical analysis of spore germination rate and spore budding tube length. Different lowercase letters indicate significant differences at P < 0.05 in Duncan’s multiple range test.

The dual culture assay approved that strain N4-3 was able to inhibit the growth of *Foc* TR4. To identify the active components with antagonistic activity, the experiment was set to determine the antagonistic activity of crude extracts of strain N4-3 and the inhibitory efficiency of volatile organic compounds against *Foc* TR4. However, no antagonistic activity of both strain N4-3 fermentation products and volatile organic compounds was detected (**Supplementary Figure 1A**, **Figure 1B**). We speculated that strain N4-3 may inhibit the growth of *Foc* TR4 by secreting enzymes with antagonistic activity. The crude enzymes were extracted using the ammonium sulfate precipitation method. Considering that the cell wall components of pathogenic fungi primarily consist of chitin and glucan (Adams, 2004; Jones et al., 2014), the activities of chitinase and *β*-1,3-glucanase were determined in crude enzyme solutions. The results showed that both chitinase and *β*-1,3-glucanase activities in the crude enzyme solution exhibited an increasing trend and then decreasing with the increase of ammonium sulfate saturation. The maximal activities were detected in the crude enzyme solution extracted with 30% of saturation of ammonium sulfate (**Figure 6A**).

To test the inhibition activity of crude enzymes against *Foc* TR4, the inhibition efficiency was determined. The results showed that the inhibition rate increased along with the increase of crude enzyme concentrations. When the concentration reached 400 mg/mL, the inhibition rate of the pathogenic mycelia was 39.96 ± 1.57% (**Figure 6B**). The EC_50_ value was 496.94 mg/mL using the toxicity regression equation. Furthermore, the effect of crude enzymes on spore germination of Foc TR4 was investigated. The germination rate of spores after treatment with EC_50_ of crude enzyme solution was only 9.98 ± 0.23% in comparison to 87.30 ± 2.03% of the control group. Additionally, the tube length of the germinated spores treated with crude enzymes was only 14.2 μm, while that in the control group reached 67.93 μm (**Figure 6C**). Therefore, crude enzyme solution not only significantly reduced the germination rate of spores, but also inhibited the length of spore germination tubes.

### Crude enzyme solution destroying the ultrastructure of *Foc* TR4 cells

**Figure 7:**
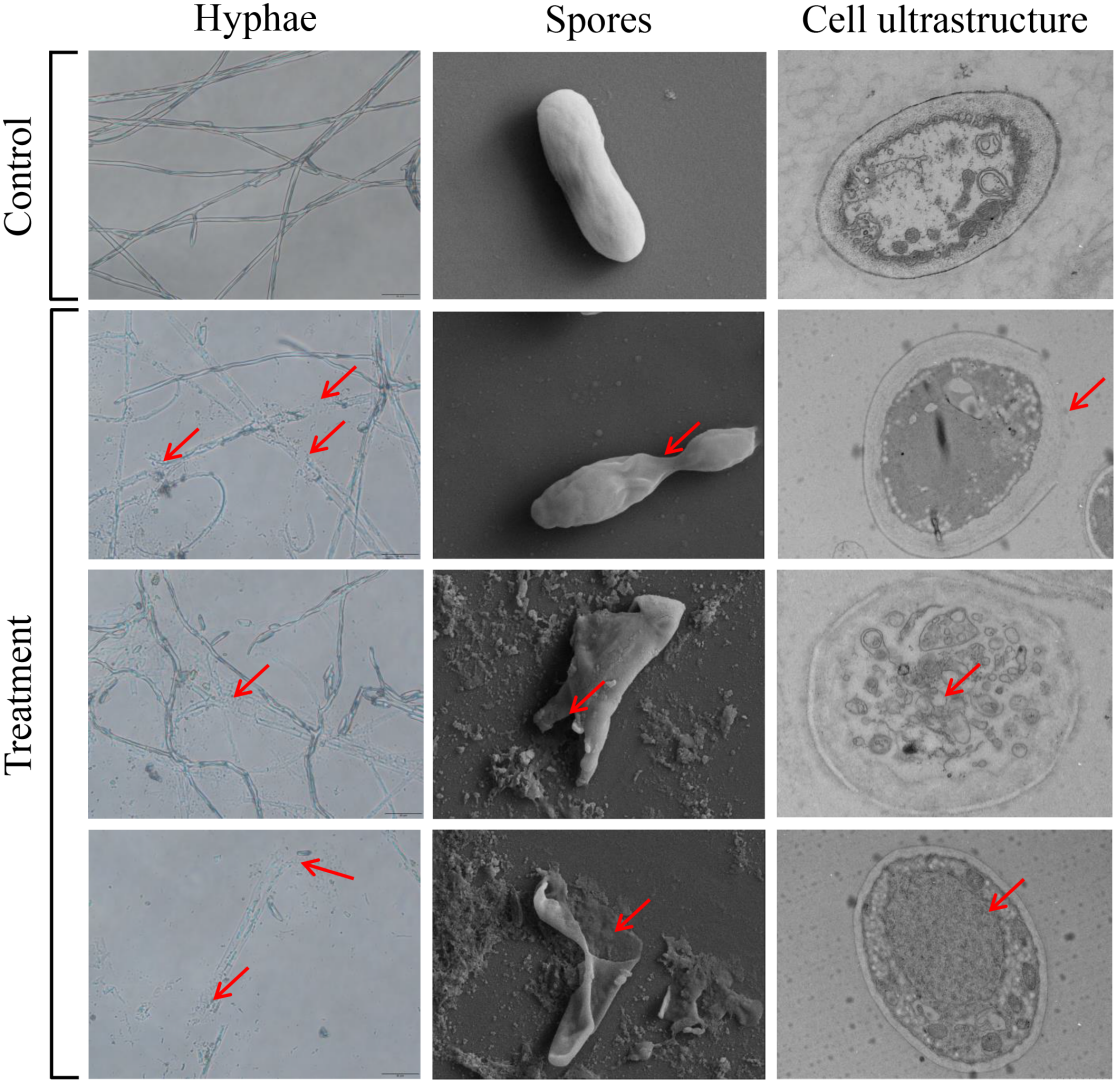
Effect of crude enzyme solution at EC_50_ concentration on *Foc* TR4 mycelium, spores and cell structure. The red arrow points to where it differs from the control.

We further evaluate whether crude enzyme solution had a disruptive effect on the morphological structure of *Foc* TR4. In the control group, the mycelial and spore morphology of the pathogenic fungi remained intact and had a smooth surface. SEM was used to observe the morphological changes of the *Foc* TR4 spores. After treatment with1×EC_50_ of crude enzymes, the mycelia of *Foc* TR4 was broken (**Figure 7**). The spores exhibited significant deformation and fragmentation, with leakage of cell contents. These observations were consistent with the results obtained from dual cultures (**Figures 5B**, **7**). SEM analysis also demonstrated intact cell walls in the normal growth of *Foc* TR4 mycelia. By contrast, cell wall of the treated group became thinner and defective. A number of intracellular vesicles and collapse of organelles were observed in *Foc* TR4 after treatment with crude enzyme solution (**Figure 7**). These results suggested that the enzymes secreted by strain N4-3 possessed antifungal activity, ultimately leading to deformation, cell wall degradation, organelle collapse and leakage of cell contents of *Foc* TR4.

### Strain N4-3 improving the resistance to *Foc* TR4 and plant growth of banana seedlings

**Figure 8:**
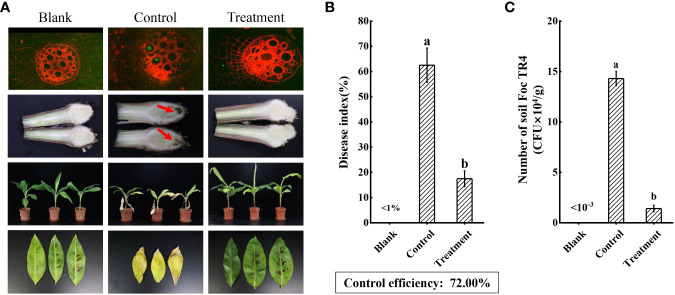
Evaluating the biocontrol effect of strain N4-3. **(A)** Growth status of banana plants in the blank (H_2_O), control (*Foc* TR4) and treatment (strain N4-3 + *Foc* TR4) at 60 dpi. The arrow indicated the site of *Foc* TR4 infection. **(B)** Statistical analysis of disease indices. **(C)** Statistical quantification of *Foc* TR4 in the rhizosphere soil of banana. The experiments were repeated three times. Different lowercase letters indicated a significant difference at the level of P<0.05.

To assess the biocontrol efficiency of strain N4-3, a pot cultivation experiment was conducted. At 60 dpi, symptoms of leaf yellowing and wilting were observed in the control plants, while no obvious disease symptom was detected in the banana plants treated with strain N4-3. Comparing with the control group, the disease index decreased from 62.50 ± 6.78% to 17.50 ± 3.12% after strain N4-3 treatment. The biocontrol efficiency was 72.00% (**Figures 8A**, **8B**). Infection of GFP-*Foc* TR4 on banana roots was observed using the laser scanning confocal microscopy. The results showed a strong green fluorescence was observed in the banana roots of the control group, while no obvious signal was detected in the treated group. Additionally, the roots of the control group exhibited black decayed symptom due to *Foc* TR4 infection (**Figure 8A**). Statistical analysis showed that the number of pathogens in the rhizosphere soil was 1.43 × 10^5^ CFU/g in the control group, while the rhizosphere soil inoculated with strain N4-3 contained 1.41 × 10^4^ CFU/g (**Figure 8C**). These results indicated that application of strain N4-3 effectively reduced the number of pathogenic fungi in the rhizosphere soil, leading to a silght symptom of *Foc* TR4 infection.

**Figure 9:**
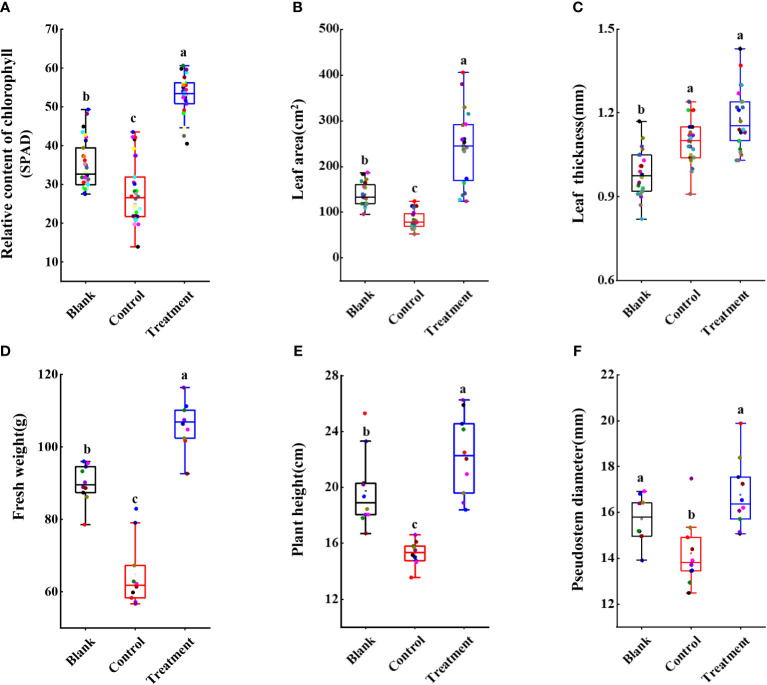
Statistical analysis of agronomic traits of banana seedlings. **(A)** Relative content of chlorophyll. **(B)** Leaf area (cm^2^). **(C)** Leaf thickness (mm). **(D)** Fresh weight (g). **(E)** Plant height (cm). **(F)** Pseudostem diameter (mm). Different lowercase letters indicate significant differences at the level of p<0.05.

Furthermore, several agronomic characteristics of banana plants were measured. At 60 dpi, the SPAD in the banana leaves of the treated group (52.12 ± 5.33) was higher than that of the control (26.88 ± 7.51) and the blank group (34.79 ± 6.22) (**Figure 9A**). Although there was no significant increase in leaf thickness between the control group (1.09 ± 0.08 mm) and the blank group (1.18 ± 0.11 mm) (**Figure 9C**), the leaf area after strain N4-3 application was 1.75-fold than that of the blank group (138.69 cm^2^) (**Figure 9B**). Moreover, the maximum photosynthetic efficiency of the leaves was also measured using the chlorophyll fluorometer. The maximum photosynthetic efficiency was 0.81 in the treated group, 0.77 in the blank group and 0.74 in control group (**Supplementary Figure 2**). These results indicated that strain N4-3 can improve photosynthesis in banana plants. Additionally, the fresh weight of the banana plants was obviously improved by strain N4-3. Compared to fresh weight in the blank group (89.90 ± 5.31 g) and the control group (64.76 ± 19.13 g) (**Figure 9D**), a significant increase was found in the treatment group (106.03 ± 6.40 g). Similar changes were observed in plant height and stem thickness (**Figures 9E**, **9F**). Hence, strain N4-3 not only improved the resistance of banana plants to *Foc* TR4, but also enhanced photosynthetic capacity and plant growth.

## Discussion

Banana Fusarium wilt caused by *Foc* TR4 is a serious disease that limits the banana industry (Kubicek et al., 2014). After successful infection of *Foc* TR4, it produces several harmful substances such as fusarium acid and beauverin, eventually leading to wilting or death of banana plants. Long-term application of chemicals can cause damage to local soil ecology (Ploetz, 2015b). Beneficial microorganisms originating from the natural environment are recognized as an important component of sustainable agriculture (Singh et al., 2020; Woo et al., 2022). Therefore, isolation and screening of beneficial microorganisms is key for the selection of biocontrol agent.

*Trichoderma* sp. is a widely utilized beneficial microorganism that exhibits a fast growth in diverse environments (Damodaran et al., 2020; Savani et al., 2021). *Trichoderma* sp. directly employs in antagonistic and competitive mechanisms to inhibit the growth of pathogenic fungi and induce systemic resistance in plants (Druzhinina et al., 2011). In our study, we aim to screen and identify one *Trichoderma* that exhibit antagonistic activity against *Foc* TR4. To isolate *Trichoderma* strains, soil samples were selected from banana plantations without disease symptom for ten years. Nine *Trichoderma* strains were isolated using the RBA medium (**Figure 1A**). By evaluating the antagonistic efficiency against *Foc* TR4, strain N4-3 had the highest antagonistic activity (**Figure 1B**). Its broad-spectrum antifungal activity was also found by a dual culture test (**Figure 4**). To further identify the strain, ML analysis based on *tef1* and *rpb2* was performed. The analysis showed that strain N4-3 clustered with *Trichoderma parareesei* CP55-3 (**Figure 2B**). The morphological characteristics of strain N4-3 supported the result (Atanasova et al., 2010). The previous by comparison of ITS sequences was normally used to identify the fungal species, but numerous fungal sister species cannot be distinguished using this method. Multiple gene sequences are concatenated to increase the number of effective informative loci, which can increase the accuracy of species identification (Heled and Drummond, 2009).

*Trichoderma parareesei* was mainly distributed in the pantropical climatic zone. It was an asexual variant of *Trichoderma reesei*. The previous study showed *Trichoderma parareesei* exhibited high parasitism and wide ecological adaptability (Herrera-Estrella et al., 2010). The rhizosphere of plants was one of the most common ecological niches for the genus *Trichoderma*. *Trichoderma parareesei* could utilize sucrose secreted by plant roots and form symbiotic interactions (Rubio et al., 2014). *Trichoderma parareesei* promoted the tolerance of *Brassica napus* L. to salt and drought by abscisic acid and ethylene singalling pathways (Poveda, 2020). Moreover, *Trichoderma* spp. could inhibit the growth of pathogenic fungi by secreting antibiotics and volatile organic compounds (Harni et al., 2020). Here, we also found that strain N4-3 exhibited a remarkable ability to inhibit the growth of *Foc* TR4 mycelia. Further experiments revealed that the metabolites and volatile organic compounds produced by *Trichoderma parareesei* N4-3 had no inhibitory actitvity on *Foc* TR4 (**Supplementary Figure 1**). By analysis, the formation of the inhibition zone may be attributed to the cell wall-degrading enzyme secreted by strain N4-3. The results were supported by that cellular structure of *Foc* TR4 was disrupted after treatment with the crude enzyme solution, resulting in the inhibition of spore germination and mycelial growth.

To further identify the important enzymes, the whole genome of strain N4-3 was sequenced. The annotation results revealed that 221 glycoside hydrolase genes were found in the genome of strain N4-3 (**Supplementary Figure 3A**). Among these, the GH18 family was responsible for the production of chitinases. *β*-1,3-glucanases synthesized by the GH16, GH55, GH64, and GH81 families played a role in degrading cell wall of pathogenic fungi (Gruber and Seidl-Seiboth, 2012). By alignment, there were 21 genes for chitinases and 26 genes for *β*-1,3-glucanases (**Supplementary Figure 3B**). This result suggested that *Trichoderma parareesei* N4-3 had the ability to secrete a large amount of cell wall-degrading enzymes to inhibit *Foc* TR4. Therefore, strain N4-3 growed rapidly to occupy the growth space and destroy the mycelia and spores of the pathogenic fungus by the production of a series of cell wall-degrading enzymes. This is a typical hyperparasitic phenomenon of *Trichoderma* (Gruber and Seidl-Seiboth, 2012).

Additionally, the abundance of *Foc* TR4 in the rhizospheric soil of banana plants after application of strain N4-3 was much lower than that in the control group (**Figure 8C**). The previous study showed that the density of pathogenic fungi was correlated with plant disease, and these fungi need to reach a certain concentration before disease appearance in plants (Zhang et al., 2021). Therefore, *Trichoderma parareesei* N4-3 could reduce the disease likelihood by inhibiting the growth of *Foc* TR4 in banana roots. Furthermore, the addition of strain N4-3 significantly increased the leaf area, relative chlorophyll content, and photosynthetic efficiency, thereby improving the resistance of plants to *Foc* TR4. Similar studies supported that beneficial microorganisms can indirectly enhance plant resistance to pathogenic fungi by promoting plant growth (Chen et al., 2020). Therefore, strain N4-3 could inhibit the growth of *Foc* TR4 through ecological niche competition and secretion of cell wall-degrading enzymes, thereby reducing the number of pathogenic fungi in the rhizospheric soil. Moreover, strain N4-3 also promoted the growth of banana plants to improve the disease resistance.

## Conclusion

In this study, *Trichoderma parareesei* N4-3 with a broad-spectrum antifungal activity was isolated and identified from banana plantation soil. *In vitro* experiments demonstrated that strain N4-3 could adhere to the surface of *Foc* TR4 hyphae. The production of chitinase and *β*-1,3-glucanase disintegrated hyphae and spores of pathogenic fungi. These enzymes degraded the cell wall, thereby disrupting the cellular structure and ultrastructure of the pathogenic fungi. The number of genes encoding chitinases and *β*-1,3-glucanase were identified in the genome of strain N4-3. In pot experiment, strain N4-3 significantly decreased the population of *Foc* TR4 in the rhizosphere of banana plants, leading to a reduction in the disease index. Therefore, *Trichoderma parareesei* N4-3 exhibited a great potential as a biocontrol agent to manage *Foc* TR4.

## Data availability statement

The original contributions presented in the study are publicly available. This data can be found here: rpb2 and tef1 sequences to GeneBank, BankIt2753675 rpb2 OR687153, BankIt2754798 tef1 OR687154, genomic data to NCBI, PRJNA1025137.

## Author contributions

WL: Conceptualization, Data curation, Investigation, Methodology, Writing – original draft, Formal Analysis, Software, Writing – review & editing. YC: Conceptualization, Data curation, Funding acquisition, Investigation, Methodology, Writing – review & editing, Software, Supervision, Writing – original draft. YW: Formal Analysis, Software, Visualization, Writing – review & editing. JF: Formal Analysis, Software, Visualization, Writing – original draft. DZ: Formal Analysis, Project administration, Software, Visualization, Writing – review & editing. BC: Formal Analysis, Software, Visualization, Writing – review & editing. DQ: Resources, Software, Validation, Writing – review & editing. MZ: Resources, Validation, Writing – review & editing. YZ: Resources, Validation, Writing – review & editing. KL: Resources, Validation, Writing – review & editing. YL: Conceptualization, Investigation, Project administration, Supervision, Writing – review & editing. WW: Funding acquisition, Investigation, Project administration, Supervision, Writing – review & editing. JX: Funding acquisition, Investigation, Project administration, Supervision, Writing – review & editing.
