# Supplementary material for: A newly isolated Trichoderma Parareesei N4-3 exhibiting a biocontrol potential for banana fusarium wilt by Hyperparasitism
Source: Front Plant Sci. 2023 Oct 24;14:1289959. doi: 10.3389/fpls.2023.1289959 (PMC10629295; doi:10.3389/fpls.2023.1289959)
Supplement: Supplementary file 6 [file DataSheet_5.docx]

Supplementary Material

# Supplementary Data

# Supplementary Figures and Tables

For more information on Supplementary Material and for details on the different file types accepted, please see [here](https://www.frontiersin.org/guidelines/author-guidelines#supplementary-material).

## Supplementary Figures


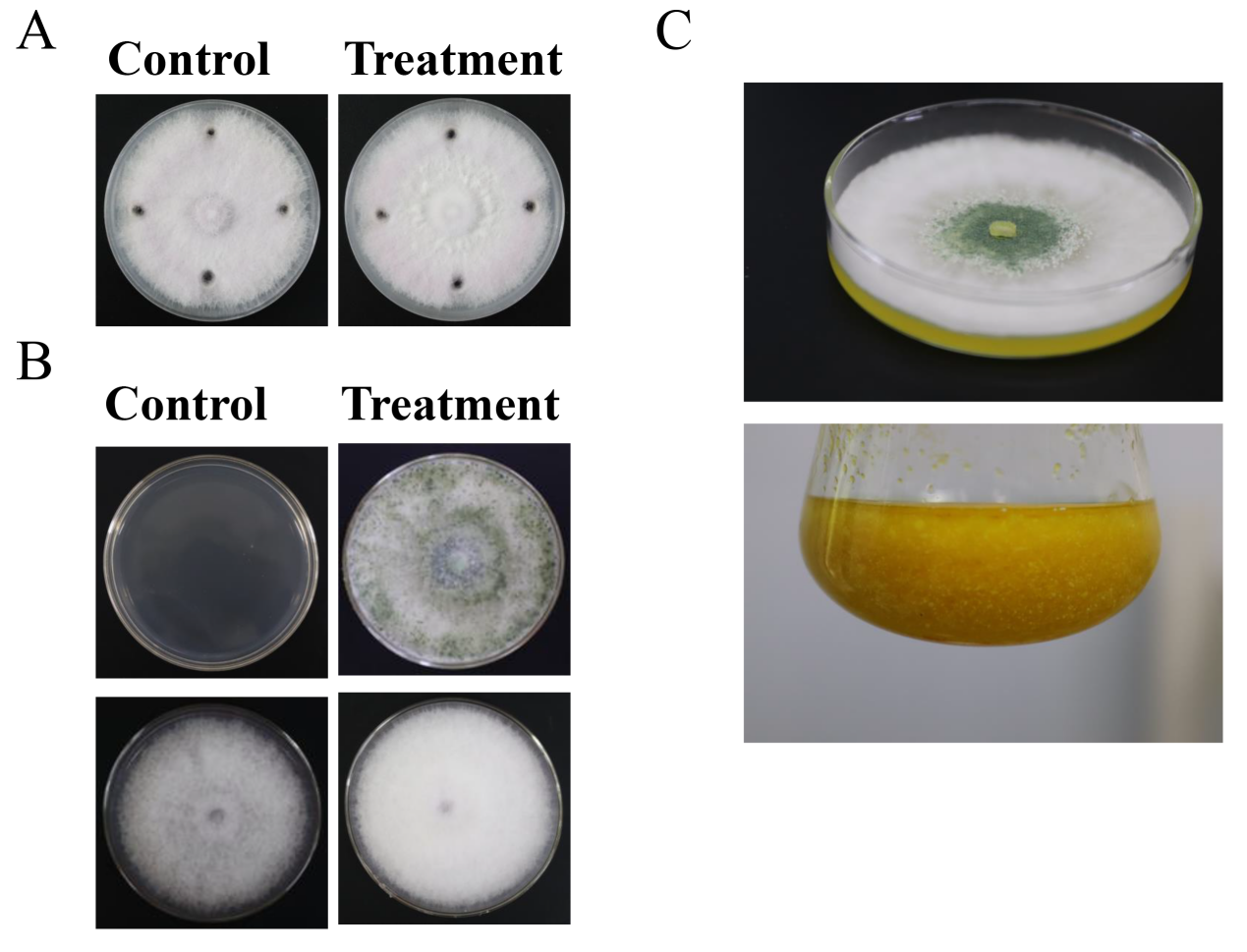


Supplementary Figure. 1 (A). Antagonistic test of N4-3 metabolites against Foc TR4. (B). antagonistic activity test of N4-3 volatile organic substances against Foc TR4. (C). growth status of N4-3 in PDA, PDB.


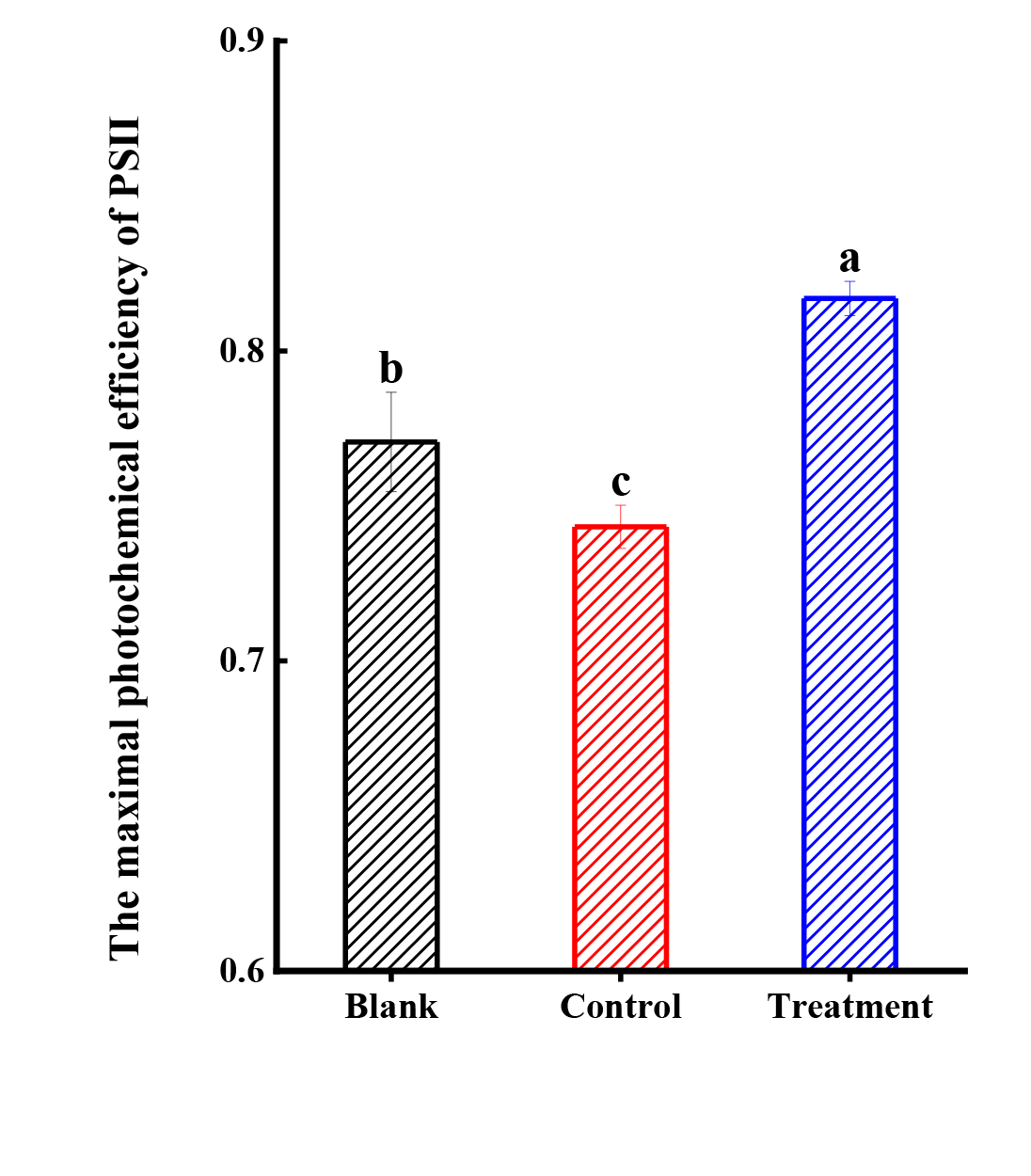


Supplementary Figure. 2. The maximal photochemical efficiency of PSⅡ.


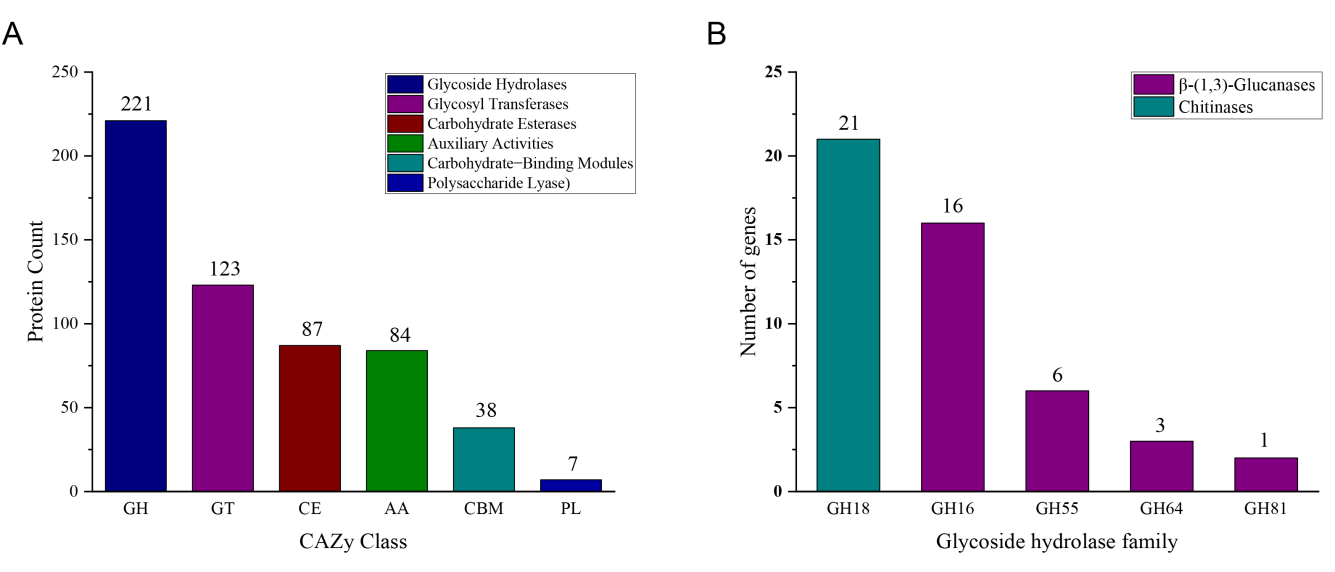


Supplementary Figure. 3. (A). CAZy functional classification statistics. (B). Number of annotations to the glycoside hydrolase family. All fungal chitinases described so far are members of GH family 18. β-1,3-glucanases have so far been described for GH families 16, 55, 64 and 81.

## Supplementary Tables

Supplementary table 1. Strains used in phylogenetic analyses and their corresponding GenBank accession numbers.

| Species | Strain | GenBank accession numbers | |
| --- | --- | --- | --- |
|  |  | tef1 | rbp2 |
| *Trichoderma parareesei* | CP55_3 | MW541129 | MW541109 |
| *Trichoderma aquatica* | YMF1.04625 | MK775507 | MK775512 |
| *Trichoderma aquatica* | YMF1.04624 | MK775506 | MK775511 |
| *Trichoderma rugosum* | 11325 | MH612379 | MH612373 |
| *Trichoderma rugosum* | 11139 | MH612378 | MH612372 |
| *Trichoderma citrinoviride* | TRS745 | KP008894 | KP009188 |
| *Trichoderma longibrachiatum* | Tloum3 | MT081437 | MT118251 |
| *Trichoderma longibrachiatum* | HL167 | MZ241241 | MZ241240 |
| *Trichoderma orientale* | S187 | JQ685868 | JQ685884 |
| *Trichoderma longibrachiatum* | TLS6 | MT069951 | MT069959 |
| *Trichoderma reesei* | CBS999.97 | CP020876.1 | CP017984.1 |
| *Trichoderma reesei* | QM6a | CP016234.1 | CP016235.1 |
| *Trichoderma breve* | T069 | OP913377.1 | OP913376.1 |
| *Trichoderma longibrachiatum* | *HL167* | MZ241241.1 | MZ241240.1 |
| *Trichoderma gamsii* | TW20050 | KU523895.1 | KU523896.1 |
| *Trichoderma capillare* | *TW22166* | KT862524.1 | KT862525.1 |
| *Trichoderma istrianum* | S310 | KJ665523.1 | KJ665281.1 |
| *Trichoderma attinorum* | LESF236 | KT279039.1 | KT278971.1 |
| *Trichoderma viridialbum* | S250 | KC285706.1 | KC285774.1 |
| *Trichoderma sempervirentis* | S599 | KC285755.1 | KC285755.1 |
| *Trichoderma pholiotae* | JZBQH12 | ON649919.1 | ON649972.1 |
| *Trichoderma istrianum* | S123 | KJ665521.1 | KJ665280.1 |
| *Trichoderma confluens* | 9649 | KT001959.1 | KT001964.1 |
| *Trichoderma viridarium* | S136 | KC285658.1 | KC285760.1 |
| *Verticillium alfalfae* | VaMs.102 | XM_003001642.1 | XM_003000681.1 |

Supplementary table 2. Carbon and nitrogen source utilization of strain N4-3.

| **Carbon source utilization** | **Result** | **Nitrogen source utilization** | **Result** |
| --- | --- | --- | --- |
| D- Mannose | +++ | ammonium chloride | ++ |
| D-Fruotose | +++ | Ammonium Sulfate | +++ |
| D-Glucose | +++ | Glycine | +++ |
| D-Melezitose | ++ | L-Arginine | - |
| D-Ribose | +++ | L-Cysteine | + |
| L-Arabinose | +++ | L-Methionine | + |
| L-Rhamnose | +++ | L-Tyrosine | ++ |
| Myo-Inositol | +++ | L-Valine | +++ |
| Raffinose | +++ | L-Hydroxyproline | + |
| Saccharose | + |  |  |
| D-Xylopyranose | ++++ | **Growth pH** | Result |
| Trehalose | +++ | 5 | _+_ |
| D-Cellose | ++ | 6 | +++ |
| Dextrin | +++ | 7 | +++ |
| D- Galactose | +++ | 8 | +++ |
| Xylan | + | 9 | + |

**Note: + + +, Good growth; ++, Moderate growth; +, Poor growth; -, No growth.**

Supplementary table 3. gene prediction stats.

| Class | Number |
| --- | --- |
| Size(base) | 32338354 |
| Protein Coding Genes | 9271 |
| Min length(base) | 120 |
| Max length(base) | 68859 |
| Average length(base) | 1522.08 |
| Total coding gene(base) | 14111190 |
| Coding ratio (%) | 43.64% |
| exon | 26026 |
| CDS | 26026 |
| Start codon | 9112 |
| Stop codon | 9003 |
| ncRNA |  |
| tRNA | 141 |
| rRNA | 46 |
| Repeat Region |  |
| Repeat Region Count | 16310 |
| Total Repeat Region(base) | 671214 |
| Repeat Ratio(%) | 2.08% |
| LINE | 84 |
| Low complexity | 1782 |
| Simple repeat | 14378 |
| Unknown | 66 |

Supplementary table 4. Genetic database annotation rate statistics.

| Database | Number of Unigenes | Percentage(%) |
| --- | --- | --- |
| Annotated in CDD | 5955 | 64.23 |
| Annotated in KOG | 4806 | 51.84 |
| Annotated in NR | 9167 | 98.88 |
| Annotated in PFAM | 4764 | 51.39 |
| Annotated in Swissprot | 6372 | 68.73 |
| Annotated in TrEMBL | 9167 | 98.88 |
| Annotated in GO | 6320 | 68.17 |
| Annotated in KEGG | 3261 | 35.17 |
| Annotated in at least one database | 9167 | 98.88 |
| Annotated in all database | 2315 | 24.97 |
| Total Unigenes | 9271 | 100 |
